# Immune dysregulation in patients with carpal tunnel syndrome

**DOI:** 10.1038/s41598-017-08123-6

**Published:** 2017-08-15

**Authors:** Gila Moalem-Taylor, Benny Baharuddin, Barbara Bennett, Arun V. Krishnan, William Huynh, Matthew C. Kiernan, Cindy Shin-Yi Lin, Boaz Shulruf, Elizabeth Keoshkerian, Barbara Cameron, Andrew Lloyd

**Affiliations:** 1 School of Medical Sciences, University of New South Wales UNSW Australia, Sydney, NSW 2052 Australia; 2 Prince of Wales Clinical School, University of New South Wales UNSW Australia, Sydney, NSW 2031 Australia; 3 Brain and Mind Centre, Sydney Medical School, The University of Sydney, and Royal Prince Alfred Hospital, Sydney, New South Wales Australia; 4 The Kirby Institute, University of New South Wales UNSW Australia, Sydney, NSW 2052 Australia

## Abstract

Peripheral immunity plays a key role in maintaining homeostasis and conferring crucial neuroprotective effects on the injured nervous system, while at the same time may contribute to increased vulnerability to neuropathic pain. Little is known about the reciprocal relationship between entrapment neuropathy and peripheral immunity. This study investigated immune profile in patients with carpal tunnel syndrome (CTS), the most prevalent entrapment neuropathy. All patients exhibited neurophysiological abnormalities in the median nerve, with the majority reporting neuropathic pain symptoms. We found a significant increase in serum CCL5, CXCL8, CXCL10 and VEGF, and in CD4+ central and effector memory T cells in CTS patients, as compared to healthy controls. CCL5 and VEGF were identified as having the highest power to discriminate between patients and controls. Interestingly, and contrary to the prevailing view of CCL5 as a pro-nociceptive factor, the level of circulating CCL5 was inversely correlated with neuropathic pain intensity and median nerve motor latency. In contrast, the level of central memory T cells was positively associated with abnormal neurophysiological findings. These results suggest that entrapment neuropathy is associated with adaptive changes in the homeostasis of memory T cells and an increase in systemic inflammatory modulating cytokines/chemokines, which potentially regulate neuropathic symptoms.

## Introduction

The immune system has increasingly been implicated in numerous neurological disorders such as neurodegenerative diseases^1^, mood disorders^2^, peripheral neuropathies and associated neuropathic pain^3, 4^. It is now well known that immune-mediated neuropathies such as Guillain-Barré syndrome (GBS), chronic inflammatory demyelinating polyneuropathy (CIDP), and multifocal motor neuropathy involve a combination of humoral and cell-mediated immune mechanisms^5–7^. Such neuropathies are believed to result from an autoimmune attack against myelin by complement-fixing autoantibodies, macrophages, and autoreactive T cells following certain bacterial or viral infections associated with molecular mimicry and loss of immune tolerance^5^. Numerous changes in the frequency of different subsets of T cells and the level of circulating cytokines have been demonstrated in peripheral blood of patients with GBS and CIDP including increased circulating CD4+ helper T (Th)1, Th17, and Th22 cells, elevated plasma levels of proinflammatory cytokines (e.g. interleukin (IL)−17)^8^, and clonal expansions in the CD8+ T cell pool, which is reflected in the clonal composition of the T cell receptor repertoire in sural nerve biopsies^9^. However, little is known about the reciprocal relationship between entrapment neuropathies, such as carpal tunnel syndrome (CTS), and peripheral immunity.

CTS is the most common peripheral nerve entrapment neuropathy reported to affect one in ten people at some point^10^. It is caused by compression of the median nerve at the wrist as it passes through the carpal tunnel leading to alterations in the endoneural blood flow, oedema formation, and ultimately ischemia and nerve injury^11–14^. CTS is characterised by sensory abnormalities including neuropathic pain symptoms such as paraesthesia and dysesthesia, numbness, tingling and hyperalgesia^10^. Neurophysiological studies have demonstrated that paroxysmal pain and abnormal sensations are correlated with demyelination of non-nociceptive Aβ fibres, whereas spontaneous constant pain is correlated with damage to nociceptive Aδ fibres^15^. Using skin biopsy in CTS patients, a recent study has shown a significant reduction in intraepidermal nerve fibre density and lengthened nodes of Ranvier in myelinated fibres indicating both small and large fibre dysfunction^16^. Quantitative sensory testing in the territory of the median nerve and in extramedian territories suggests the involvement of both peripheral and central sensitisation in neuropathic pain associated with CTS^17^. Over the past two decades, the immune system has been recognised as a major contributor to neuropathic pain both at the peripheral and central nervous system^3, 18^. In particular, animal models of partial peripheral nerve injury or progressive mild nerve compression have demonstrated a significant local and remote immune-mediated inflammation including recruitment and activation of macrophages and peripheral blood mononuclear cells and T cell infiltration into the injured nervous system along the sensory neuraxis^19–22^. As such, the present study sought to investigate whether changes in peripheral immunity were manifested in patients with painful peripheral neuropathy.

Cell-mediated immunity involves the production of cytokines and chemokines (chemotactic cytokines) in response to an antigen and is mediated by T cells. The acquisition of antigen experience is manifested by the generation and persistence of long-lived memory T cells^23^. Recent studies have demonstrated an alteration in the balance of different T cell subtypes in patients with chronic low back pain and neuropathic pain with a significant increase in the frequency of anti-inflammatory regulatory T (Treg) cells^24, 25^. However, little is known about the immune profile in patients with peripheral neuropathy. To this end, studies were undertaken in patients diagnosed with CTS as a well-defined model of idiopathic localised peripheral entrapment neuropathy. Specifically, the present study evaluated neuropathic pain symptoms, neurophysiological changes in the median nerve, and characterised the expression of circulating cytokines and chemokines and the profile of systemic T cell subsets, with a focus on memory T cells and Treg cells.

## Results

### Demographic and clinical data

The demographic and clinical details of the 26 patients and 26 control subjects recruited for the study are shown in Table 1. The majority of CTS patients (76%) were females, with a mean age of 56 years, in accordance with previous studies^16, 26^. The body mass index (BMI) of all subjects was calculated based on their reported height and weight. BMI of CTS patients was significantly higher than control subjects, reflecting the fact that a higher BMI is considered to be a risk factor for CTS^27^. There were no differences between the groups in caffeine consumption, smoking or exercise frequency. However, a significantly higher level of distress was observed in the CTS patients, as indicated by significantly higher scores of depression, anxiety and stress.

**Table 1:** Demographic and clinical data.

	Carpal tunnel syndrome (n = 26)	Control subjects (n = 26)	*p* value
Gender (male/female)	5/21	8/18	0.45^
Age (years) (mean; SD)	56.4 (10.1)	54 (10.5)	0.39
Country of birth Australia - n (%)	11 (42)	11 (42)	
Marital Relationship – partnered -n (%)	19 (73)	20 (79)	
	**Mean (SD)**		
Body Mass Index (BMI)	29 (8)	25 (3)	0.05
Caffeine consumption -cups/day	3.0 (2.6)	3.4 (2.1)	0.53
Number cigarettes/day	1.5 (5.7)	0	0.10
Exercise - hours/week	5.5 (9.7)	5.0 (5.2)	0.84
Moderate exercise/week - hours	4.9 (8.0)	2.9 (2.4)	0.22
Vigorous exercise/week - hours	0.6 (1.5)	1.1 (1.7)	0.24
***DASS***_**21**_ **Subscales**	**Mean (SD)**		
Depression	3.54 (3.70)	1.77 (2.66)	0.05
Anxiety	2.50 (1.90)	0.73 (1.11)	<0.001
Stress	5.54 (4.05)	3.38 (2.73)	0.03

SD = standard deviation; DASS21 = Depression, Anxiety, Stress scale 21 item; ^Chi-square test.

The characteristics of the pain symptoms in CTS patients are shown in Table 2. The symptoms were present on both sides in 65% of patients, and only on the right side or left side in 27% and 8%, respectively. Patients rated their pain in the hand using the numeric rating scale ranging from 0 (no pain) to 10 (most severe pain) with a mean score of 3.8, and varied symptom duration ranging from 8 weeks to 6 years. Of the 26 patients with CTS, the Douleur Neuropathique 4 (DN4) questionnaire identified a majority of 23 patients (88%) with neuropathic pain. Neuropathic pain symptom inventory (NPSI) analysis of five distinct dimensions of pain demonstrated that these patients had burning pain, deep pressing pain, paroxysmal pain and evoked pain with a mean rating of 2.4–2.9 out of 10 each, and paraesthesia/dysaesthesia with a mean rating of 5.5 out of 10.

**Table 2:** Patient’s symptoms.

Affected limb(s)	(n; %)
Bilateral	17 (65)
Right	7 (27)
Left	2 (8)
Numeric Pain Rating Scale (range 1–10) (mean; SD)	3.8 (2.56)
DN4 score (mean; SD)	5.1 (1.5)
**NPSI (pain type) subscale scores (range 0–10** **=** ***dimensions*** **of pain)**	**Mean (SD)**
Burning pain	2.85 (3.20)
Deep spontaneous pain	2.44 (2.63)
Paroxysmal pain	2.63 (3.18)
Evoked pain	2.47 (2.94)
Paresthesia/dysesthesia	5.52 (3.44)
Total pain *intensity* score (range 0−100)	31.46 (25.59)

DN4 = Douleur Neuropathique 4 - Neuropathic Pain questionnaire; NPSI = Neuropathic Pain Symptom Inventory.

Table 3 summarises the neurophysiology data of patients with CTS. Comparison of the neurophysiological parameters of the 52 hands tested confirmed abnormal median sensory and motor response in affected limbs with 62.8% mild, 23.3% moderate, and 13.9% severe changes. Nerve conduction studies showed a significant decrease in the median sensory nerve action potential (SNAP) amplitude, sensory nerve conduction velocity, and the compound muscle action potential (CMAP) amplitude, and a significant increase in distal motor latency in affected versus unaffected limbs. No changes were observed in the ulnar SNAP amplitude and sensory nerve conduction velocity, which was within normal limits in all hands tested (mean > 50 m/s). These neurophysiology data confirm median nerve entrapment neuropathy.

**Table 3:** Neurophysiology data of patients with carpal tunnel syndrome.

	Affected Limbs (n = 43); Mean (SD)	Non-affected limbs (n = 9); Mean (SD)	*P*-value
**Median Nerve**			
Sensory amplitude (μV)	7.48 (4.95)	16.87 (7.02)	0.004
Sensory conduction velocity (m/s)	36.62 (37.70)	52.77 (4.80)	0.001
Motor amplitude (mV)	7.00 (2.50)	9.81 (2.10)	0.006
Distal motor latency (ms)	4.38 (1.12)	3.18 (0.45)	<0.001
**Ulnar Nerve**			
Sensory amplitude (μV)	8.11 (3.97)	9.63 (1.97)	NS
Sensory conduction velocity (m/s)	53.33 (4.76)	53.21 (2.86)	NS

μV = microvolt; m/s = meter/second; mV = millivolt; ms = millisecond; NS = not significant.

### Patients with carpal tunnel syndrome have a significant increase in distinct serum cytokine profiles

The family of cytokines plays a crucial role in signalling network between cells and in regulating both the immune and nervous systems. Both cytokines and chemokines have been implicated in modulating neuronal excitability and contributing to neuropathic pain^28^. We therefore tested serum levels of 27 cytokines/chemokines using a human cytokine multiplex assay. The levels of IL-1β, basic fibroblast growth factor (bFGF), chemokine (C-C motif) ligand 3 (CCL3; also known as macrophage inflammatory protein 1-alpha - MIP-1α), granulocyte-macrophage colony-stimulating factor (GM-CSF) and IL-15 were undetectable and were therefore excluded. All other markers were sorted into the following three functional groups:

1. Chemokines: CCL4, also known as macrophage inflammatory protein-1β (MIP-1β); CCL5, also known as RANTES (regulated on activation, normal T cell expressed and secreted); Eotaxin; C-X-C motif chemokine 10 (CXCL10), also known as interferon gamma-induced protein 10 (IP-10); CCL2, also known as monocyte chemoattractant protein-1 (MCP-1); and CXCL8, also known as IL-8.
2. Cytokines: IL-6; Interferon gamma (IFNγ); IL-1 receptor antagonist (IL-1RA); IL-5; Tumour necrosis factor (TNF)α; IL-2; IL-13; IL-4; IL-10; IL-7; IL-12; IL-17A; and IL-9.
3. Growth/Stimulating factors: vascular endothelial growth factor (VEGF); Platelet-derived growth factor (PDGF); and Granulocyte-colony stimulating factor (G-CSF).

**Figure 1:**
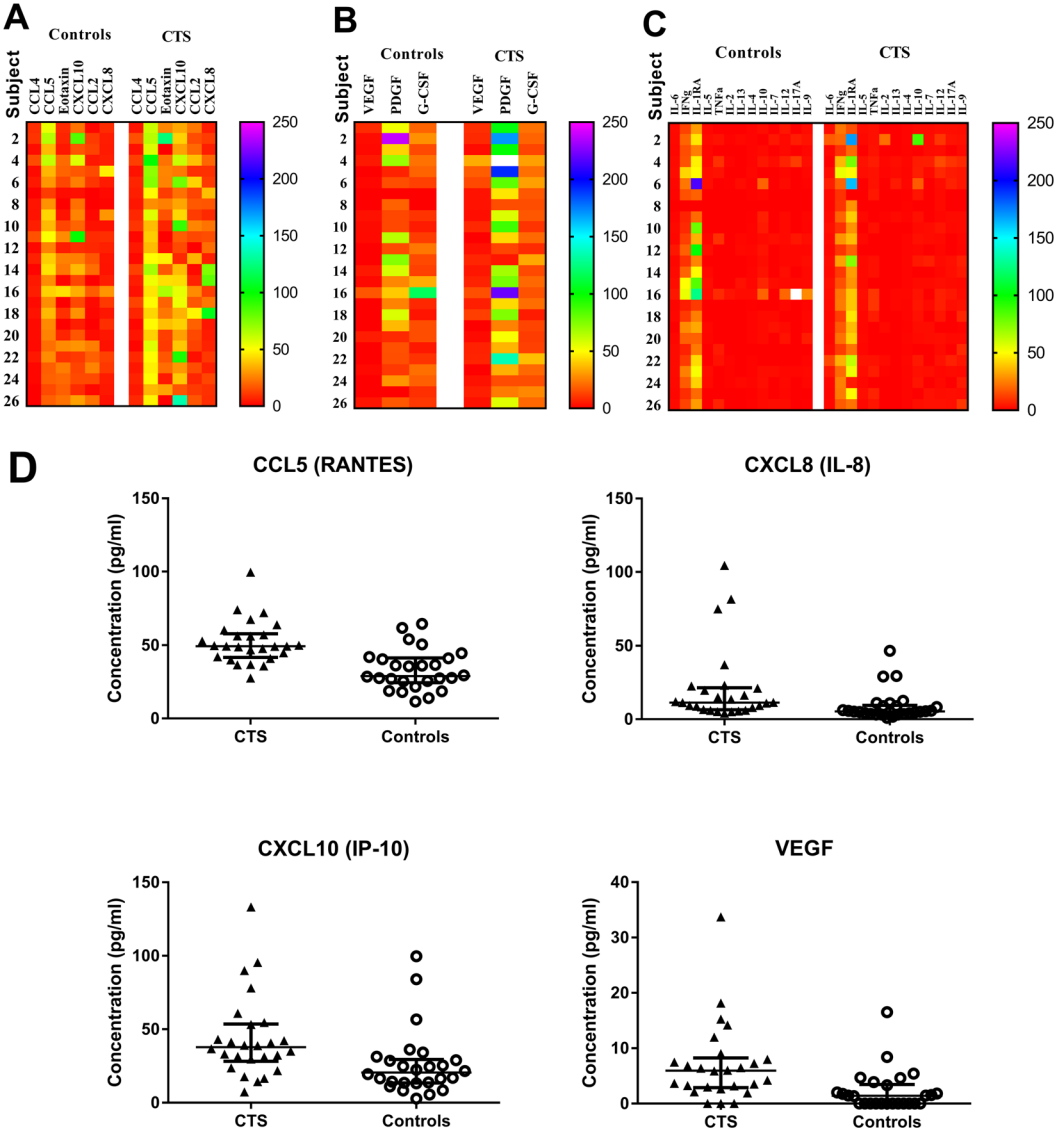
Serum cytokine/chemokine levels in CTS patients. Heat maps comparing the expression profile of control and CTS serum cytokines, which are grouped as (**A**) Chemokines; (**B**) Growth factors; and (**C**). Cytokines. Values are median cytokine concentration (pg/ml), with red indicating lowest levels, blue-purple indicating highest levels, and yellow-green indicating median levels. (**D**) Serum CCL5, CXCL8, CXCL10 and VEGF levels are significantly increased in CTS patients (n = 26) relative to healthy controls (n = 26). Lines represent the median and interquartile range.

Heatmaps depicting the relative levels of chemokines (Fig. 1A), stimulating/growth factors (Fig. 1B) and cytokines (Fig. 1C) in CTS patients and controls show generally higher levels of serum chemokines and growth factors than cytokines in all subjects with clear upregulation in CTS patients. Table 4 shows a comparison of the median and 95% confidence interval (CI) of the cytokine, chemokine and growth factor data in CTS patients and control subjects with the significance levels analysed by Mann-Whitney U test. The levels of VEGF, CCL5, CXCL8 and CXCL10 retained significance (*P* < 0.0023) after Bonferroni correction for multiple comparisons, with significantly elevated levels in CTS patients compared to controls (Fig. 1D).

**Table 4:** Serum chemokines, cytokines, and growth/stimulating factors.

Marker	Controls	CTS	*P*-value
**Chemokines**			
CCL4 (MIP-1β)	2.79 (1.60–4.67)	5.03 (3.74–7.49)	0.0369
CCL5 (RANTES)	28.89 (25.57–40.38)	49.28 (44.87–56.25)	0.000035*
Eotaxin	11.46 (5.49–20.76)	20.07 (11.61–25.70)	0.1011
CXCL10 (IP-10)	20.53 (13.84–28.54)	37.76 (31.2–43.11)	0.0010*
CCL2 (MCP-1)	7.83 (1.4–14.2)	14.78 (11.5–20.26)	0.0100
CXCL8 (IL-8)	5.33 (4.12–8.91)	11.37 (7.95–19.72)	0.0009*
**Cytokines**			
IL-6	1.42 (1.16–1.85)	1.7 (1.37–2.23)	0.080
IFNγ	15.2 (11.15–19.37)	19.84 (12.04–24.07)	0.113
IL-1RA	33.79 (24.69–49.32)	37.89 (28.7–49.32)	1.000
IL-5	1.07 (0–1.63)	1.53 (1.16–2)	0.179
TNFα	0 (0–3.33)	2.89 (0–3.79)	0.066
IL-2	0 (0–0)	0 (0–0)	0.788
IL-13	0 (0–0)	0 (0–0)	0.782
IL-4	0.58 (0.45–0.72)	0.71 (0.63–0.84)	0.025
IL-10	1.82 (1.49–3.22)	2.91 (1.62–3.86)	0.372
IL-7	0 (0–1.57)	1.64 (0–2.21)	0.095
IL-12	1.4 (1.03–1.79)	2.11 (1.59–3.12)	0.013
IL-17A	0 (0–1.03)	1.89 (0–2.93)	0.005
IL-9	1.15 (0.76–1.56)	2.11 (1.56–4.03)	0.005
**Growth/Stimulating factors**			
VEGF	1.41 (0–2.03)	5.94 (3.19–7.49)	0.00025*
PDGF	22.18 (14.34–41.14)	56.60 (35.11–87.77)	0.00383
G-CSF	13.31 (9.95–19.41)	18.09 (14.16–21.19)	0.05107

Data are presented as median (pg/ml) and 95% confidence interval. *P*-values calculated using Mann–Whitney U test. *Denotes significance following Bonferroni correction for multiple comparisons (*P* < 0.0023).

Because CTS patients showed both increased levels of certain cytokines/chemokines and increased BMI and negative emotional states (depression, anxiety and stress), we performed partial correlation analysis to assess the independent association between these parameters. For this purpose, the correlation between the cytokines VEGF, CCL5, CXCL8 and CXCL10 and BMI, depression, anxiety and stress was assessed while controlling for the neuropathy. The results showed no statistically significant correlations (*P* > 0.10) for both controlled and uncontrolled correlation analyses, suggesting that the increased levels of serum cytokines/chemokines are independent of BMI and negative emotional states.

Next, we used discriminant function analysis (DFA) to identify selected variables within each cytokine group that best discriminate between patients and controls. Such analysis enables identifying groups of markers that alterations in their levels can define a profile and create a diagnostic pattern in specific condition. CTS patients were readily distinguished from controls based on their chemokine (CCL4, CCL5, CXCL10, CCL2, and CXCL8) profile with 80.8% classification accuracy (Gamma = 0.893, *P* < 0.00001; Kappa = 0.615, *P* < 0.00001) and their growth/stimulating factor (VEGF, PDGF, and G-CSF) profile with 69.2% classification accuracy (Gamma = 0.703, P < 0.002; Kappa = 0.385, P < 0.004). The cytokines IL-4, IL-12, IL-17A and IL-9 were also able to discriminate CTS patients and controls, but with lower classification accuracy. Further analysis to determine the best independent discriminators between CTS patients and controls using DFA with stepwise method revealed that CCL5 levels alone predicted CTS with 76.9% accuracy (Gamma = 0.839, P < 0.0001; Kappa = 0.38, P < 0.0001) and VEGF levels alone predicted CTS with 69.2% accuracy (Gamma = 0.835, P < 0.004; Kappa = 0.385, P < 0.001). Thus, CCL5 and VEGF were considered as the best discriminatory cytokines to distinguish between CTS patients and healthy controls.

### T cell phenotyping in patients with carpal tunnel syndrome shows an increased frequency of CD4+ memory T cells

**Figure 2:**
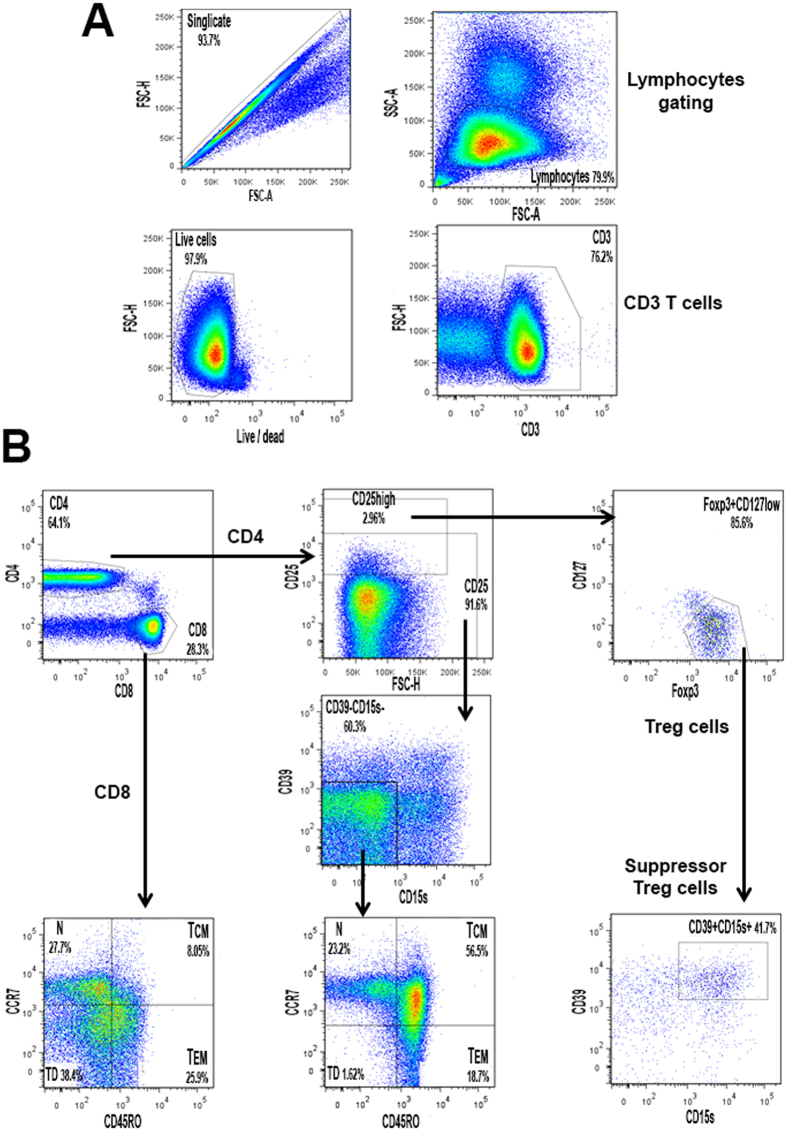
Gating strategy for T cell phenotyping in PBMCs. (**A**) Flow cytometry to identify T cell subsets in PBMCs was carried out in CTS and control subjects. Singlet cells were first gated, followed by gating of lymphocytes according to their size (forward-scatter) and granularity (side-scatter). Live cells were distinguished from dead cells using a staining kit (Live/dead UV450) and then gated for CD3+ cells. (**B**) CD3+ cells were differentiated into CD4+ and CD8+ T cells and further into naïve or memory cells. After antigen experience, memory T cells gain expression of CD45RO and lose expression of CD45RA. Thus, CD45RO was used to differentiate the naïve from memory populations. CC-chemokine receptor 7 (CCR7) divides human memory T cells into two functionally distinct subsets: Effector memory T (T_EM_) cells that express low levels of CCR7 but have receptors for migration to inflamed tissues and display immediate effector function, and central memory T (T_CM_) cells that express high levels of CCR7 that enable them to access and enter the lymph nodes from blood. CD8+ T cells were differentiated into subpopulations by dividing CD45RO *vs* CCR7 into quadrants (naïve-N, central memory-﻿T_CM_, effector memory-T_EM_ and terminally differentiated-TD). CD4+ T cells were gated for CD25 high population, CD127 low and Foxp3+ cells to identify regulatory T (Treg) cells. Treg cells were then further classified as suppressor Treg cells by double positive staining of CD39 and CD15s (sialyl Lewis x). Non Treg CD4+ cells were then differentiated into subpopulations by dividing CD45RO *vs* CCR7 into quadrants (naïve-N, central memory-T_CM_, effector memory-T_EM_ and terminally differentiated-TD).

**Figure 3:**
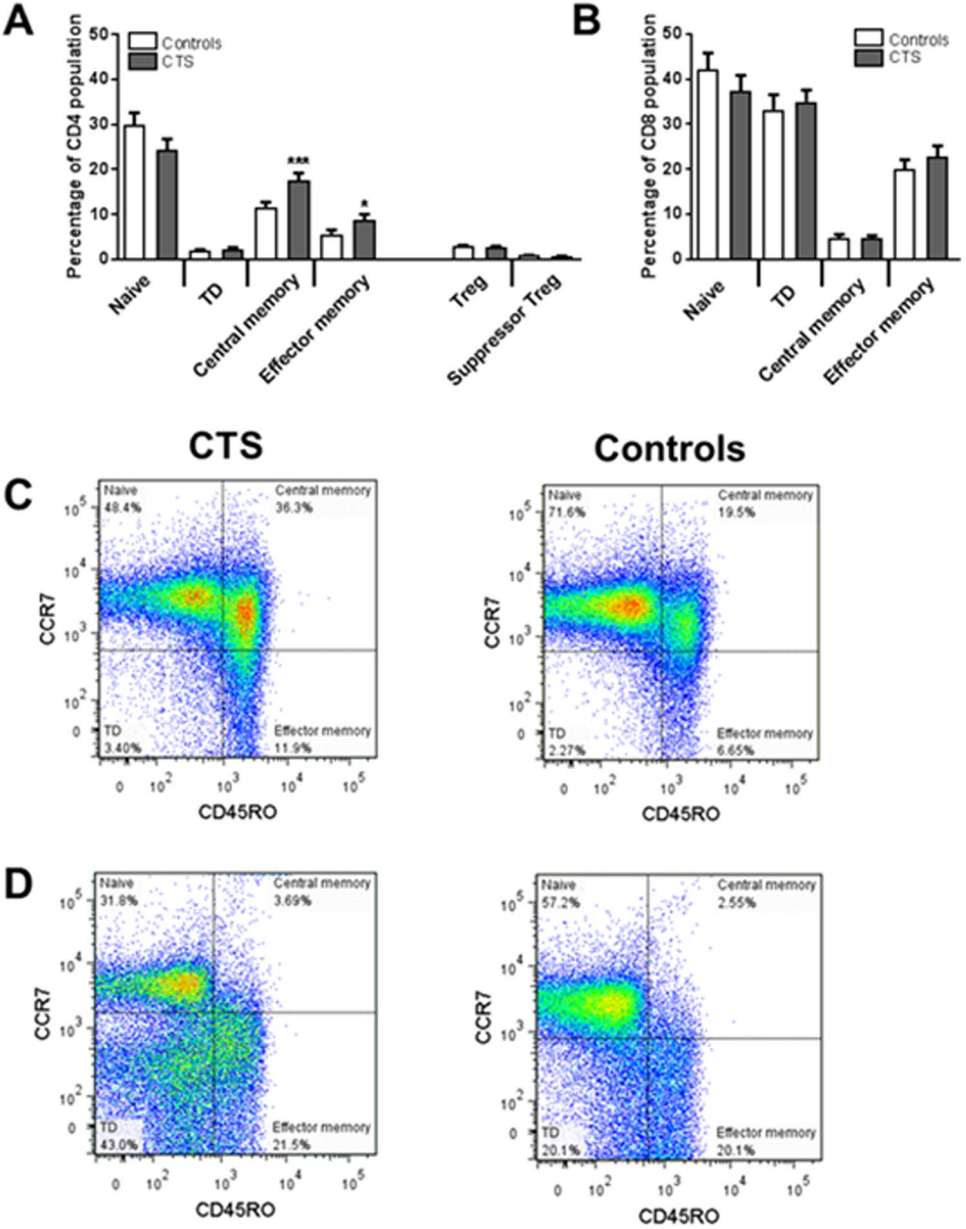
Increased CD4+ memory T cells in patients with CTS. Naïve and memory T cell subsets in PBMCs of patients with CTS (n = 26) and normal controls (n = 26) were identified by the expressions of CD45RO and CCR7 after gating on CD4+ or CD8+ T cells in PBMCs. CD4+ Treg cells were identified by the expression of CD25 high, CD127 low and Foxp3+. Supressor Treg cells were further identified by double positive staining of CD39 and CD15s. (**A**) Percentages of naïve, terminally differentiated (TD), central memory and effector memory in CD4+ T cells, and CD4+ Treg cells and supressor Treg cells. (**B**) Percentages of naïve, TD, central memory and effector memory in CD8+ T cells. Columns represent mean + SEM. *P < 0.05; ***P < 0.005. Representative flow cytometry scatter plots are shown for (**C**) CD4 and (**D**) CD8 memory T cell populations for CTS patients (left panel) and control subjects (right panel).

Because some serum cytokines/chemokines were significantly increased in CTS patients, we examined whether there are changes in circulating immune cells. White blood cell count showed no difference between CTS patients and healthy controls in the absolute numbers (mean ± SD; million per ml) of leukocytes (CTS: 5.98 ± 2.81; Controls: 5.6 ± 1.99), lymphocytes (CTS: 1.86 ± 0.73; Controls: 1.87 ± 0.73), monocytes (CTS: 0.30 ± 0.21; Controls: 0.25 ± 0.10), and neutrophils (CTS: 3.86 ± 1.99; Controls: 3.52 ± 1.42). To further assess T cell subpopulations in peripheral blood mononuclear cells (PBMCs), we designed a flow cytometric assay that identifies the prevalence of CD4+ and CD8+ memory T cell subsets, as well as total Treg cells (CD4+, CD25high, CD127low and Foxp3+) and suppressive Treg cells based on their CD15s^29^ and CD39^30^ expression (Fig. 2). Our analysis showed that compared to healthy controls, CTS patients have a significant increase in the frequency of CD4+ central memory (CCR7+ CD45RO+) T cells (P = 0.0042) and effector memory (CCR7-CD45RO+) T cells (P = 0.048) (Fig. 3A,C), with no change in the frequency of total or suppressive Treg cells (Fig. 3A) or in CD8+ naïve, effector, and memory cells (Fig. 3B,D).

### IFN-γ responses to neurofascin and peripheral myelin P2 proteins did not change in carpal tunnel syndrome patients

**Figure 4:**
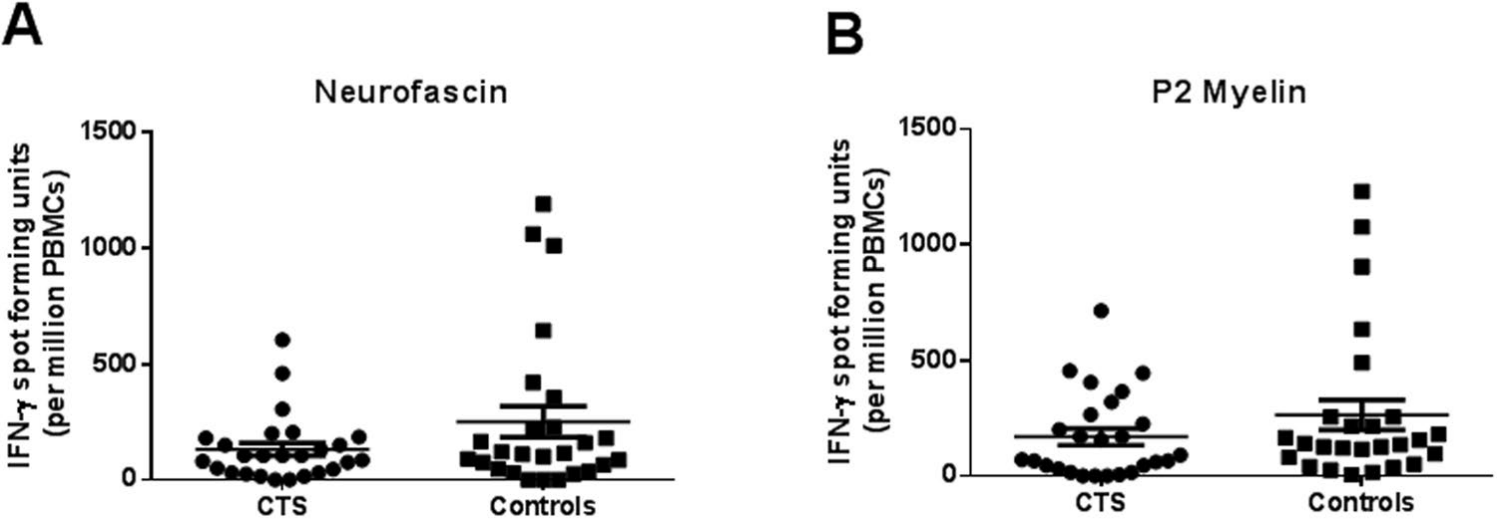
Absence of difference in the level of neurofascin/P2-specific T cells between CTS patients and healthy controls. ELISPOT assay was used for detection of interferon (IFN)-γ-secreting cells following stimulation with peripheral myelin antigens in PBMCs from CTS patients (n = 26) and normal controls (n = 26). There was no difference between groups in the total number of IFN-γ spots per million cells in response to neurofascin (**A**) and P2 myelin (**B**). Data are expressed as mean + SEM.

Since an increase in antigen-experienced memory CD4+ T cells was observed in our CTS patients and since increased T cell reactivity to peripheral myelin proteins has been reported in patients with immune-mediated neuropathies^31, 32^, we next assessed the frequency of antigen specific cytokine secreting PBMCs using an enzyme-linked immunospot (ELISPOT) assay. CTS patients and healthy controls were tested for the detection of IFN-γ+ memory T cells specific for myelin P2 recombinant protein and recombinant human neurofascin protein. Positive control wells stimulated with a non-specific stimulus (CD3 beads) showed very high IFN-γ responses (>500 spots/well) in all CTS and control samples. Figure 4 depicts the frequency of IFN-γ+ spot forming cells in the PBMCs stimulated with either neurofascin (Fig. 4A) or myelin P2 (Fig. 4B) protein. There were no differences in spot frequencies between CTS patients and healthy controls.

### Correlation of immune markers with patients’ neuropathic pain scores and neurophysiology

**Figure 5:**
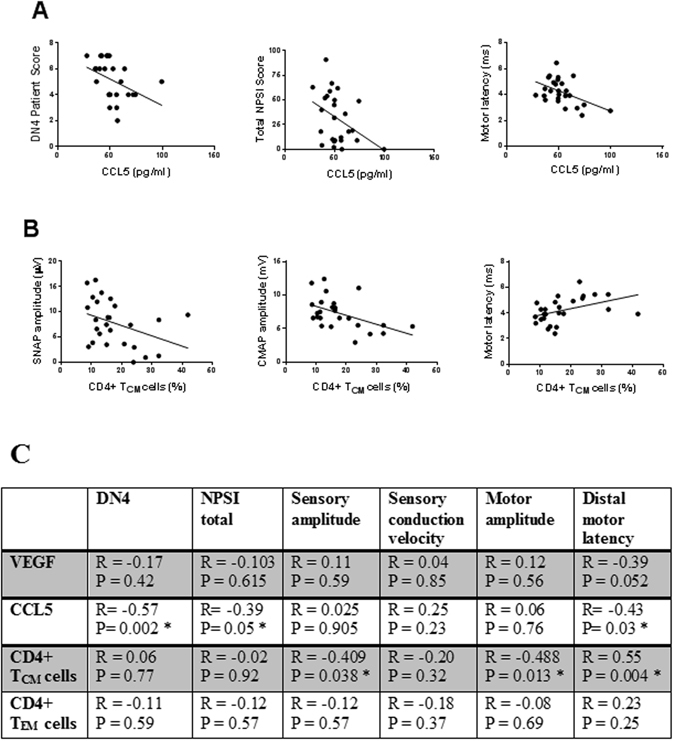
Correlations between immune markers, neuropathic pain scores, and neurophysiological parameters. (**A**) Serum CCL5 is inversly correlated with the neuropathic pain scores of DN4 and total NPSI, and with distal motor latency. (**B**) The percentage of central memory CD4+ T cells (T_CM_) is inversly correlated with the SNAP and CMAP amplitudes, and positively correlated with the distal motor latency. Spearman’s rank correlation coefficient was used for analysis. (**C**) A summary of Spearman correlation coefficients (P-values) of VEGF, CCL5, and memory T (T_CM_ and T_EM_﻿) cells ﻿﻿﻿﻿with patient’s neuropathic pain symptoms and neurophysiological parameters. *Denotes significant correlation coefficients.

As CCL5 and VEGF were determined as the most discriminatory variables between CTS patients and healthy controls, we further tested their correlation with patient neuropathic pain intensity and neurophysiological parameters. There was no correlation between VEGF levels, patient pain symptoms and neurophysiological results. Surprisingly however, the levels of CCL5 were inversely correlated with the DN4 (r = −0.57; *P* = 0.002) and total NPSI (r = −0.39; *P* = 0.05) scores. In addition, CCL5 levels were also inversely correlated with the median nerve distal motor latency (r = −0.43; *P* = 0.03) (Fig. 5A).

The levels of both effector and central memory T cells, which also showed a significant increase in CTS patients, did not correlate with any of the patients’ pain symptoms. Interestingly however, the percentage of central memory T cells was negatively correlated with both the median nerve SNAP and CMAP amplitudes (r = −0.409, *P* = 0.038; r = −0.488, *P* = 0.013, respectively), and positively correlated with the distal motor latency (r = 0.55, *P* = 0.004) (Fig. 5B).

Figure 5C summarises the results of the correlation coefficients of neuropathic pain scores and neurophysiological parameters with the levels of VEGF, CCL5, CD4+ central memory T cells, and CD4+ effector memory T cells in CTS patients.

## Discussion

Dysregulation of cytokines and altered immune responses have been associated across a range of neurological conditions, including neuropathic pain^3, 4, 24, 28^. Here, through the analysis of blood samples from patients with CTS and associated neuropathic pain, we found that several specific serum cytokines, particularly chemokines, were significantly increased as compared to healthy controls. Unexpectedly, one of these chemokines, CCL5, was found to be inversely correlated with the intensity of neuropathic pain. In addition, a significant increase in the percentage of both central and effector memory CD4+ T cells was observed in the PBMCs of CTS patients as compared with controls. Levels of CD4+ central memory T cells were associated with neurophysiological data of median nerve abnormalities. These findings support the notion of disturbed T cell homeostasis and cytokine/chemokine imbalance in neuropathic pain. However, whether such changes directly contribute to the pathophysiology of neuropathy or are merely an indirect consequence of other mechanisms underlying CTS remain unknown. Indeed, there is a complex interaction between pain and emotional (depression, stress and anxiety) states, and metabolic and immune functions, which can contribute to higher circulating levels of inflammatory mediators^2, 4^. Although we haven’t found a significant correlation between the BMI, depression, anxiety, and stress and the increased cytokine levels, we cannot exclude the possibility of an indirect influence of these variables on immune dysregulation in CTS.

Several mechanisms have been implicated in CTS including intraneural ischemia with subsequent formation of oedema, Schwann cell reactivity and demyelination followed by ectopic impulse generation, and central sensitisation^16, 17^. Recently however, neuroinflammation has been suggested as a potential contributor to the pathogenesis of CTS^33^. An animal study using a model of entrapment neuropathy with mild peripheral nerve compression in rats showed chronic neuroinflammation with macrophage and T cell invasion both locally at the compression site in the nerve and in related dorsal root ganglia. These inflammatory responses were associated with the development of neuropathic pain symptoms including mechanical hyperalgesia and cold allodynia^21^. The expression of cytokines and chemokines is a prominent feature of neuroinflammation and elevated levels of certain cytokines, such as TNFα, IL-6, and IL-1β, have been demonstrated in patients with painful peripheral neuropathies, such as GBS and CIDP^34–39^.

The assessment of multiple cytokines and chemokines revealed a dysregulated pattern of neuropathy-specific cytokine profile in CTS. In particular, the growth factor VEGF and the chemokines CCL5, CXCL8 and CXCL10 were significantly increased in CTS patients as compared to healthy controls. Furthermore, the serum cytokine profiles of CTS patients were readily distinguishable from those of controls with distinct patterns of the chemokines CCL2, CCL4, CCL5, CXCL8 and CXCL10 and the growth factors VEGF, PDGF and G-CSF, which may be induced by the neuropathy. These sets of CTS specific markers indicate that there is interplay of a broad spectrum of immunomodulatory proteins that drive the inflammatory process in this neuropathy. Another important aspect of our findings is that a stepwise DFA of cytokine data was able to effectively establish the diagnostic capability of CCL5 and VEGF that could best discriminate between CTS patients and healthy controls. If confirmed in other studies, this tool may prove to be a useful biomarker for determining the diagnosis and potential prognosis of entrapment neuropathy, and may improve the specificity and sensitivity of intervention strategies in the clinical setting^40^.

VEGF is a key regulator of physiological vasculogenesis and angiogenesis, and also promotes neurogenesis, neuroprotection and glial growth in the nervous system^41^. Accumulating evidence from experimental models of chemotherapy-induced peripheral neuropathy^42, 43^, ischemic peripheral neuropathy^44^ and diabetic neuropathy^45^, which all commonly share reduced endoneurial blood flow and nerve hypoxia, suggests a protective role for VEGF. VEGF gene therapy in these animal models of neuropathy resulted in restoration of nerve blood flow, improvement in nerve electrophysiology by slowing the development of reduced nerve conduction velocities and inhibition of axonal degeneration^42–45^. Since VEGF is induced by hypoxia^46^, it is logical to assume that the increased levels of circulating VEGF in CTS patients reflect its production due to hypoxia following median nerve compression, and it may serve an endogenous protective role. VEGF signalling has also been demonstrated to contribute to neuropathic pain symptoms as it is upregulated in injured nerves and potentiates pain transmission in dorsal root ganglia and spinal cord neurons after peripheral nerve injury in rodents^47, 48^. Thus, whereas the increased serum VEGF may be pro-angiogenic and neuroprotective, it may also be pro-nociceptive after median nerve compression in CTS.

Chemokines are a group of structurally related molecules that are divided into two major subfamilies based upon cysteine residues position, CXC and CC, and regulate cell trafficking of various types of leukocytes^49^. For example, CCL2, CCL4, and CCL5 are all chemotactic for monocytes, both CCL2 and CCL5 additionally recruit memory T cells^50, 51^, and CCL4 preferentially induces migration of naive T cells^52^ to lymph nodes and sites of inflammation. CXCL8 is a potent chemoattractant for neutrophils^53^, and CXCL10 is a potent chemoattractant for effector T cells^54^. Previous studies have shown an increase of both CXC and CC chemokines in the cerebrospinal fluid and/or serum of patients with GBS and CIDP^34, 55, 56^ suggesting their involvement in the extravasation of blood‐derived inflammatory leukocytes into the neural parenchyma. In addition, both CXC and CC chemokines have been shown to play a role in the pathogenesis of neuropathic pain, particularly by modulating the electrical activity of neurons and driving sustained excitability of primary afferent and secondary neurons within spinal pain pathways^57^. Hence, our findings of increased chemokine expression in CTS patients suggest they may play a role in recruitment of leukocytes to the inflamed tissue, and potentially in directly or indirectly mediating neuropathic pain symptoms.

Investigating the relationship between the variables using correlation analyses surprisingly revealed an inverse correlation between CCL5 levels and neuropathic pain scores, and the median nerve motor latency in CTS patients. A recent human study has demonstrated a significant increase in CCL5 in the jawbone of patients with atypical facial pain and trigeminal neuralgia^58^, but no evidence was presented for its role in mediating pain in these neuropathic pain patients. Yet, numerous animal studies using CCL5 knockout mice or pharmacological blocking of CCL5 have shown that CCL5 mediates an inflammatory response in the injured nerve and glial activation in the spinal cord, thereby facilitating neuropathic pain^59–62^. Contrary to these studies, our results suggest that although serum CCL5 is significantly increased in CTS patients, its high levels are associated with reduced neuropathic pain. In addition, higher CCL5 levels were associated with lower median nerve distal motor latencies in the CTS patients having prolonged motor latencies. The distal motor latency represents the time required for conduction through the distal axons, the neuromuscular transmission time, and the time needed to generate a muscle action potential, and it is altered in the distal median nerve segment in CTS reflecting an abnormal motor response^63^. This negative correlation suggests that CCL5 may be protective. Indeed, besides being pro-inflammatory, CCL5 and its receptors are also expressed in the nervous system, and CCL5 has been shown to play a neuroprotective role both *in vitro* and *in vivo* in several models of neuronal injury^64–67^. While the cellular source of CCL5 in our study is unclear, CCL5 can potentially be produced not only by lymphocytes and macrophages that may be activated by the nerve damage, but also by endothelial cells, glia and neurons. Proposed mechanisms for its neuroprotective effects are activation of Akt and Erk1/2, pro-survival molecules for neurons, attenuating the cleavage of caspase-3, a key mediator of apoptosis, and inducing the expression of neurotrophic factors, such as brain-derived neurotrophic factor, in neuronal cells^66^. Thus, it is possible that CCL5 plays a dual role in promoting both inflammation and neuroprotection following nerve damage, thereby regulating neuropathic pain.

Chemokines play a well-established role in recruitment of naïve, effector, and memory T cells. Activated naive T cells differentiate into terminal effector T cells that die, and into a diverse array of memory T cells that persist in the host^23^. Using flow cytometric analysis, we quantified the frequency of naïve, tissue-homing effector memory T (T_EM_) cells that are capable of immediate effector functions, and lymph-node-homing central memory T (T_CM_) cells that are capable of migrating to secondary lymphoid organs and stimulating a second wave of T cell response. In addition, since CD4+ Treg cells are indispensable for immunological self-tolerance and homeostasis, we quantified the frequency of total Treg cells and suppression-competent Treg cells. Interestingly, we found a significant increase in both CD4+ T_EM_ and T_CM_ cells in the PBMCs of CTS patients compared to controls. The upregulation of memory T cells may suggest the presence of a persistent antigenic stimulus in the nerve-affected subjects and may reflect a chronic inflammatory response. Indeed, perturbation in the homeostasis of memory T cells has been reported in several diseases^68–70^. Interestingly, T_CM_ levels were associated with abnormal neurophysiological findings in the median nerve suggesting a positive correlation between T_CM_ numbers and the severity of median nerve compression. T_CM_ cells are the predominant cell type in the human cerebrospinal fluid^71^. It is thus possible that circulating T_CM_ cells monitor the nervous system, retaining the capacity either to initiate local immune reactions in the damaged nerve and related dorsal root ganglia, or to return to secondary lymphoid organs. We found no corresponding increase in the number of interferon-γ secreting T cells following stimulation with neurofascin and peripheral myelin P2 protein, and consequently the antigen specificity of these antigen-experienced T cells remains unknown. Potential antigens that may drive the conversion of naïve cells into CD4+ memory T cells^72^ following the nerve damage in CTS are other myelin proteins and nodal antigens, such as myelin basic protein, myelin protein P0 and Contactin-1^6^.

Treg cells have recently been implicated in endogenous recovery from neuropathy-induced pain in animal studies^73, 74^ and their number was found to increase in mixed population of patients with peripheral neuropathic pain (symmetrical polyneuropathy/peripheral mononeuropathy), postherpetic neuralgia, and orofacial pain^24^. In contrast, our results showed no change in total Treg or suppressive Treg cell frequency between CTS patients and controls suggesting that T cell imbalance may be specific for each condition. While this study revealed an increased frequency of circulating CD4+ memory T cells, future studies will investigate whether other peripheral immune cells, such as B cell subsets, and anti-neuronal antibodies are also altered in CTS patients.

In conclusion, we show for the first-time dysregulated patterns of neuropathy-specific cytokine/chemokine profile and an altered phenotype of circulating CD4+ T cell subpopulations with enhanced CD4+ memory T cell frequency in CTS patients. In addition, we demonstrate significant negative correlations between the levels of circulating CCL5, neuropathic pain intensity and distal median nerve latency and positive correlations between the levels of CD4+ memory T cells and abnormal neurophysiological findings. Our results suggest the presence of an ongoing antigenic stimulus resulting in expanded memory T cell population and upregulation of specific cytokines due to the nerve compression in CTS patients. This immune dysregulation might contribute to the maintenance of the condition that may persist after the initial episode of acute inflammation has been resolved. However, the antigenic specificity of these antigen-experienced T cells remains unknown. In addition, the expanded memory T cell subsets and the upregulated chemokines can be pro-inflammatory and pro-nociceptive, and/or neuroprotective with anti-nociceptive effects in neuropathic pain. Lastly, these results need to be interpreted with caution due to the low number of subjects and the fact we cannot determine whether such changes in systemic immune markers are the cause or consequence of the entrapment neuropathy and associated neuropathic pain.

## Methods

### Study design and subjects

Between 2013 and 2015, 26 patients with CTS confirmed by electrodiagnostic and clinical criteria were recruited at the Department of Neurology, Prince of Wales Hospital, where they underwent neurophysiological testing followed by questionnaire assessments for pain symptoms and general health and wellbeing, as well as blood sampling. Patients were excluded if they had potentially confounding conditions such as pregnancy, an autoimmune or inflammatory disease (e.g. multiple sclerosis, rheumatoid arthritis), an active infection (e.g. HIV/AIDS, hepatitis), neurological conditions (e.g. head injury, radiculopathy, fibromyalgia) or cancer, and if they were taking immunosuppressive oral medications. In addition, 26 age and gender matched pain-free healthy volunteers without any confounding medical condition were included. The study was approved by the South Eastern Sydney Area Health Service and University of New South Wales Human Research Ethics Committee. All participants provided written informed consent, and all methods were performed in accordance with the relevant guidelines and regulations.

### Patient’s assessment and neurophysiological examination

All subjects were assessed for relevant demographic, medical history and life style information. They were then given several questionnaires: (1) A 0–10 pain intensity Numeric Rating Scale (NRS) for scoring the severity of current pain; (2) The DN4 questionnaire for assessing the probability of neuropathic pain^75^, which includes limited physical examination; (3) Patients who scored ≥4 on the DN4, completed the NPSI questionnaire for clinical symptoms of constant burning (superficial) pain, constant pressing (deep) pain, paroxysmal pain, provoked pain (allodynia, hyperalgesia) and abnormal sensations (paresthesia and dysesthesia)^76^; (4) Depression, Anxiety and Stress Scales (DASS21), which assesses a range of symptoms common to depression and anxiety, comorbidities in neuropathic pain.

Clinical diagnosis of CTS was based on diagnostic criteria of the American Academy of Neurology (AAN)/American Association of Neuromuscular and Electrodiagnostic Medicine (AANEM). These included nocturnal and/or activity-related sensory symptoms, sensory deficits in median nerve distribution, and weakness and/or atrophy of median-innervated muscles^77^. Electrophysiological studies were conducted with a NicoletTM EDX device (Natus, CA, USA), using standard techniques based on AANEM practice parameters^77^. All studies were conducted at standard room temperature. Orthodromic recording of the median nerve SNAP was obtained following stimulation of the index finger using ring electrodes. The onset latency, peak amplitude, and sensory conduction velocity (SCV) were assessed. The median nerve CMAP was recorded from abductor pollicis brevis following stimulation of the nerve at the wrist. The distal motor latency (DML) and peak amplitude of the CMAP were also measured. Ulnar nerve was stimulated at the fifth digit, with recordings obtained at the wrist. For motor studies, the nerve was stimulated at the wrist and a motor potential was recorded from abductor digit minimi. In some patients, differential sensory studies were carried out. Our laboratory reference values were: median SCV > 50 m/s; median nerve DML < 4.0 ms; median-to-radial sensory (thumb) latency difference < 0.5 ms; and median-to-ulnar (fourth digit) sensory difference < 0.5 ms^78^.

### Blood sampling and processing

Peripheral blood was collected from patients into acid citrate dextrose (ACD) Vacutainers and into clotting (no anticoagulant) tubes (Vacuette, Greiner bio-one, Australia). Complete blood counts including white cell differential were performed using an automated COULTER Ac.TTM diff Haematology Analyser (Beckman Coulter, Australia). Serum was separated and stored in aliquots at −80 °C. PBMCs were separated by Ficoll-Hypaque density gradient centrifugation and cryopreserved in Roswell Park Memorial Institute (RPMI) medium (Sigma-Aldrich, Australia) with 10% dimethyl sulfoxide (DMSO; Sigma) and 50% autologous plasma. PBMCs aliquoted into Cryovials (Nunc CryoTubes, Thermo Fisher, Australia) were first frozen by placing in a CoolCell (Biocision, USA), in a −80 °C freezer overnight to allow a decrease in temperature by 1 °C/min, and finally were stored in vapour phase liquid nitrogen. Before assays, frozen PBMCs were thawed rapidly in a 37 °C water bath. Cells were washed twice with 10 ml pre-warmed RPMI medium, counted, and cell density was then adjusted to the desired concentration with RPMI.

### Cytokine/chemokine multiplex assay

Sera were thawed and analysed undiluted following the instructions for the Bio-Plex Pro Human Cytokine 27 Assays kit (Bio-Rad, USA). Standards were made up following the manufacturer’s recommendations, and run in duplicate. The 96 well filter plate was pre-wet with BioPlex assay buffer and washed using a magnetic wash station with blotting after every wash. Coupled beads (1x) were added to the assay plate and washed twice without removing the beads. 50 µl of samples and standards were then added to the relevant wells and incubated for 30 minutes, covered with foil at room temperature on a shaking platform. The plate was then washed three times with BioPlex wash buffer and detection antibody added to all wells. The plate was then incubated for 30 minutes in the dark on a shaking platform. Streptavidin-PE (1x) was added to all wells, incubated for 10 minutes in the dark on a shaking platform. The plate was then washed three times and beads were resuspended in assay buffer, before analysis on a Magpix using the xPONENT software (Luminex, USA). Background values were subtracted from sample readings, and sample concentrations that were lower than background or below detection limit were assigned a value of zero. Individual analytes were excluded from analysis if the number of values assigned as zero equalled or exceeded 80% of total number of values. One data point identified as a clear outlier was removed from the data (in IL-17A).

### Flow cytometry

PBMCs were suspended in 1000 µl of wash buffer (PBS+ 0.5%BSA) and stained with 1 µl of reconstituted Live/Dead fluorescent reactive dye (Life Technologies, Australia) to distinguish live cells, as per the manufacturer’s instructions. The following antibodies were used to label surface antigens for the primary T cell populations: CD3 APC-H7, CD4 FITC and CD8 AF700 (BD Biosciences, Australia). To identify T cell subpopulations, CD45RO BV605 and CCR7 V450 stained for naïve, central memory, effector memory and terminally differentiated cells. In addition, CD25 APC and CD127 BV421 (BD Biosciences, Australia) were used to identify Treg cells, and CD39 PE-Cy7 (Biolegend, Australia) and CD15s BV711 (BD Biosciences, Australia) were used to identify suppressor Treg cells. Subsequently, cells were fixed and permeabilised for intracellular staining (Foxp3/transcription factor staining buffer set, eBioscience, Australia) with Foxp3 PE antibody, used as a Treg cell marker in conjunction with CD4 and CD25.

Flow cytometric acquisition after compensation acquired at least 100,000–200,000 events using a LSRFortessa flow cytometer with FACSDiva software (BD Biosciences, USA).

Flow cytometry analysis was carried out using FlowJo software (Tree Star Inc, USA), as in Fig. 2. Singlet cells were defined as lymphocytes according to their size (FSC) and granularity (SSC). Live cells were selected from FSC vs Live/dead UV450, then gated for CD3^+^ cells, and differentiated into CD4^+^ and CD8^+^ T cells. CD4^+^ T cells were gated for CD25 high population, CD127 low and Foxp3^+^ cells to identify Treg cells, which were then further classified as suppressor Treg cells by double positive staining of CD39^79^ and CD15s (sialyl Lewis x). CD15s has recently been identified as a highly specific surface marker for activated and most suppressive Foxp3 (high) effector Treg cells, which is able to differentiate them in various clinical settings from non-suppressive Foxp3+ T cells in humans^29^. Both CD8+ T cells and non Treg CD4+ cells were further differentiated into subpopulations by dividing CD45RO vs CCR7 into quadrants (naïve, central memory, effector memory and terminally differentiated).

### ELISPOT assay

PBMCs were thawed and adjusted to 2 × 10^6^ cells/ml in culture media (10% FBS) and rested overnight in a 50 ml tube at 37 °C, 5% CO_2_. IFN-γ ELISPOT assay was performed using the human IFN-γ ELISPOT^basic^ ALP kit (Mabtech, Germany). A 96-well, nitrocellulose-base plate (Multiscreen, Millipore, USA) was pre-wet with sterile 35% molecular grade ethanol and washed three times in PBS. The plate was coated with capture anti-human IFN-γ monoclonal antibody (2.5 µg/ml) and incubated overnight at 4 °C.

The coated plate was washed six times with PBS before blocking for two hours with RPMI containing 5% foetal bovine serum (FBS). Cell number was adjusted using RPMI (20% FBS) to a final concentration of 4 × 10^6^ cells/ml. Blocking media was tipped off before adding PBMCs (2 × 10^5^/well) and stimulants: CD3 beads (cell:bead concentration of 1:1) as a positive control, myelin P2 recombinant protein (Novus Biologicals, CO, USA) and recombinant human neurofascin protein (R&D Systems, Inc., MN, USA) at 50 μg/ml, and media alone as negative control. All conditions were tested in triplicate. PBMCs were incubated for 24hr at 37 °C, 5% CO_2_. PBMCs and media were tipped off before washing with PBS and incubating with biotinylated anti-human IFN-γ monoclonal antibody (1 µg/ml) for two hours. The plate was washed six times before incubating with streptavidin-alkaline phosphatase (Sigma) for 1 hr. After washing, the plate was incubated with bromochloroindolyl phosphate/nitroblue tetrazolium substrate (BPNT) (Sigma) for at least 20 min. The colour reaction was stopped by washing the plate in water before removing the plate backing, and then left to dry overnight. Spots were counted using a computer-assisted ELISPOT analyzer (Autoimmun Diagnostika). Results were calculated as the mean of triplicate wells, expressed as the number of IFN-γ spot forming cells per 1 × 10^6^ cells, with the background counts subtracted.

### Statistical analysis

Statistical analyses were performed using SPSS Statistics (version 22, IBM, NY USA) and NCSS 11 Data Analysis (Kaysville, Utah, USA). Graphs were created using GraphPad Prism Software (version 6.0, GraphPad, CA, USA) for Windows. For normally distributed data, a two-tailed *t* test was carried out to compare different parameters between CTS patients and controls. For non-normally distributed data, the non-parametric Mann-Whitney U-test was used. P values of < 0.05 were considered to be statistically significant. Results are expressed as mean ± standard deviation/error or median and interquartile range, as indicated. For analysis of cytokine data, a Bonferroni adjustment to correct for multiple comparisons, based on 22 markers, was used and the significance level for difference between groups was adjusted to *P* < 0.0023. DFA was used to classify CTS patients and controls based on a distinct cytokine profile. A stepwise DFA was then used to identify the markers that best discriminate between patients and controls. Correlations between specific cytokine and chemokine levels, on the one hand, and neuropathic pain scores and neurophysiological data, on the other hand, were calculated using Spearman’s rank correlation. The independent association between the significant cytokines and BMI, depression, anxiety and stress was carried out using partial correlation analysis controlled for health status.
